# Resonant MEMS Accelerometer with Low Cross-Axis Sensitivity—Optimized Based on BP and NSGA-II Algorithms

**DOI:** 10.3390/mi15081049

**Published:** 2024-08-18

**Authors:** Jiaqi Miao, Pinghua Li, Mingchen Lv, Suzhen Nie, Yang Liu, Ruimei Liang, Weijiang Ma, Xuye Zhuang

**Affiliations:** College of Mechanical Engineering, Shandong University of Technology, Zibo 255000, China; 13581071927@163.com (J.M.); llipinghua@sdut.edu.cn (P.L.); w2764699195@163.com (M.L.); szhen0203@163.com (S.N.); 13287887673@163.com (Y.L.); lrm25790@163.com (R.L.); 18894377579@163.com (W.M.)

**Keywords:** MEMS resonant accelerometer, BP and NSGA-II algorithm, cross-axis sensitivity, multi-objective optimization

## Abstract

This article proposes a low cross-axis sensitivity resonant MEMS(Micro-Electro-Mechanical Systems) accelerometer that is optimized based on the BP and NSGA-II algorithms. When resonant accelerometers are used in seismic monitoring, automotive safety systems, and navigation applications, high immunity and low cross-axis sensitivity are required. To improve the high immunity of the accelerometer, a coupling structure is introduced. This structure effectively separates the symmetric and antisymmetric mode frequencies of the DETF resonator and prevents mode coupling. To obtain higher detection accuracy and low cross-axis sensitivity, a decoupling structure is introduced. To find the optimal dimensional parameters of the decoupled structure, the BP and NSGA-II algorithms are used to optimize the dimensional parameters of the decoupled structure. The optimized decoupled structure has an axial stiffness of 6032.21 N/m and a transverse stiffness of 6.29 N/m. The finite element analysis results show that the sensitivity of the accelerometer is 59.1 Hz/g (*Y*-axis) and 59 Hz/g (*X*-axis). Cross-axis sensitivity is 0.508% (*Y*-axis) and 0.339% (*X*-axis), which is significantly lower than most resonant accelerometers. The coupling structure and optimization method proposed in this paper provide a new solution for designing resonant accelerometers with high interference immunity and low cross-axis sensitivity.

## 1. Introduction

Micro-Electro-Mechanical Systems (MEMS) technology has grown rapidly over the past few decades and is widely used in fields such as inertial navigation [1,2,3], consumer electronics [4], medical devices [5,6], and wireless communications [7]. As an important branch of MEMS technology, MEMS silicon-based resonant accelerometers (SRAs) have performed well in the field of acceleration measurement due to their advantages of quasi-digital output, high accuracy, high stability, and miniaturization [8,9]. The SRA is used to measure the acceleration of an object and convert it into a number and are widely used in navigation, tilt measurement [10], vibration monitoring, and seismic detection [11]. In these applications, the cross-axis sensitivity and stability of the SRA are key performance indicators that directly affect measurement accuracy and system reliability. Lower cross-axis sensitivity significantly improves system performance and ensures system stability and safety. Therefore, there are strict requirements for cross-axis sensitivity in the design of SRAs. In automotive airbag systems, cross-axis sensitivity is usually required to be less than 5% [12]. In seismic monitoring and navigation applications, cross-axis sensitivity is required to be less than 1%. The high stability of SRAs can provide consistent and accurate measurement results under complex conditions, which reduces errors and signal noise, thereby improving the overall performance and reliability of the system.

Double-ended tuning fork (DETF) resonators have a central role in SRAs since the symmetric and antisymmetric modes of the DETF resonator are adjacent modes and resonate similarly (a difference of a few thousand Hz). When the DETF resonator operates in symmetric modes, it is susceptible to antisymmetric mode interference, which can lead to energy dissipation, thus affecting the quality factor (Q) and stability of the device. To address this challenge and enhance the symmetry of the operating modes in the resonator face, Han et al. proposed a DETF resonator with a coupling structure in the design of resonant pressure sensors, which enables the DETF to operate in symmetric modes, away from antisymmetric modes, and improves the Q value [13]. Liu et al. designed a piezoelectric (DETF) resonator with dual-drive capability based on aluminum nitride (AlN). The design of dual-drive electrodes can improve the symmetry of the resonator in the operating mode and increase the Q value [14]. However, the one proposed by Han et al. is mainly applied to resonant pressure sensors, while the one used by Liu et al. has limitations when applied to silicon-based resonant accelerometers due to its complex processing. Therefore, to better improve the performance of silicon-based SRAs, new solutions are needed to overcome these challenges and improve the performance and stability of the devices.

In addition, to improve the detection accuracy of SRAs with crosstalk between the *X*-and *Y*-axis, a dual-axis MEMS resonant accelerometer was designed by Yang et al. A U-beam was used as the decoupling structure. The *X*-axis and *Y*-axis sensitivities of this accelerometer are 52.57 Hz/g and 51.64 Hz/g. The *X*-axis cross-axis sensitivity is 1.08%, and the *Y*-axis cross-axis sensitivity is 1.33% [15]. Ding et al. similarly designed a biaxial resonant MEMS accelerometer with a decoupled structure, using an H-beam as the decoupled beam. The sensitivity of this accelerometer was 275 Hz/g, and the cross-axis sensitivity was less than 3.4% [16].

For performing multi-objective parameter optimization, the NSGA II optimization algorithm has powerful global and local search capabilities for complex multi-objective optimization problems. It can efficiently handle nonlinear, degree, and multi-constraint optimization tasks with diverse and stable solution sets, providing a set of uniformly distributed Pareto optimal solutions [17]. Therefore, the NSGA II optimization algorithm is suitable for application in MEMS resonant sensors and is usually combined with other algorithms. Lv et al. used the BP and NSGA-II optimization algorithms in the design of MEMS resonant pressure sensors with high sensitivity and wide range [18]. Zhang et al. used the BP and NSGA-II optimization algorithms in the design of optical resonant cavities to optimize the quality factor and sensitivity of the device, which provided a new optimization for the design of runway ring resonant cavities [19].

To more effectively use resonant MEMS accelerometers in seismic monitoring, automotive safety systems, and navigation, DETF resonators with coupled structures are used for the first time in SRAs, as described in this paper. By introducing the coupling structure, the frequency difference between the symmetric and antisymmetric modes of the resonator is effectively improved to avoid Q loss so that the stability of the accelerometer is effectively improved. In addition, to address the problem of high cross-axis sensitivity in most of the current resonant accelerometers, the dimensional parameters of the decoupling structure are optimized for the first time using the BP-NSGA II optimization algorithm to reduce the *X*-axis *Y*-axis crosstalk during the design of resonant MEMS accelerometers. The optimized decoupling structure has a good decoupling effect that reduces the cross-axis sensitivity of the accelerometer to 0.508% (*Y*-axis) and 0.339% (*X*-axis). It can effectively meet the needs in the fields of seismic monitoring, automotive safety systems, and navigation. This study provides strong technical support for the follow-up research of SRAs.

## 2. Principle and Design of SRAs with Coupled Structure

**Figure 1:**
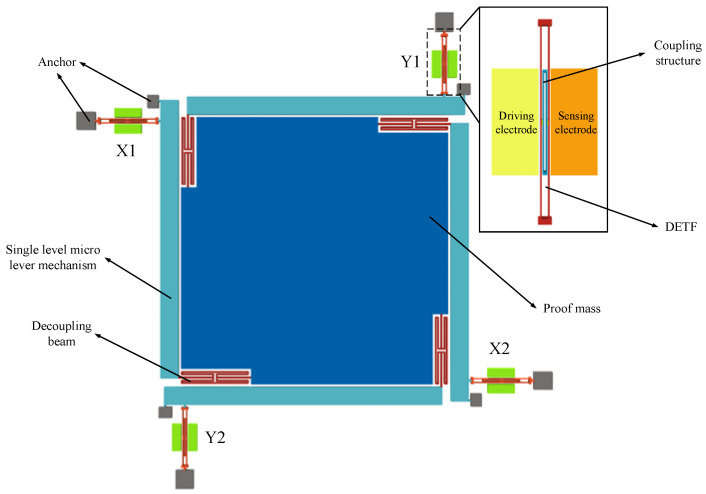
Schematic diagram of the overall structure of an SRA with a coupled structure.

The overall structure of the SRA with the coupled structure designed in this paper is shown in Figure 1. The SRA consists of a proof block and four identical sensing mechanisms. Each sensing mechanism consists of a decoupling structure, a single-stage microlever mechanism, and a DETF resonator with a coupling structure. The excitation and detection mechanism of the SRA consists of a DETF resonator with a coupling structure, as well as drive electrodes and sense electrodes provided on the left and right sides. For proper operation of the resonator, a certain DC bias voltage is usually applied to the resonator, and an AC drive voltage is applied to the drive electrode. A parallel plate capacitor is formed between the electrodes and the resonant beam. Due to the electrostatic force, the resonator will resonate when the electrostatic driving force is intrinsically the same as that of the resonator, resulting in the resonant beam vibrating periodically with the driving voltage. On this side of the sensing electrode, a varying capacitance is formed due to periodic variations in the gap between the vibrating resonant beam and the sensing electrode. Since the resonator beam is biased with a voltage, an induced current appears at the sense electrode, and the vibration state of the resonator can be detected by detecting the induced current.

**Figure 2:**
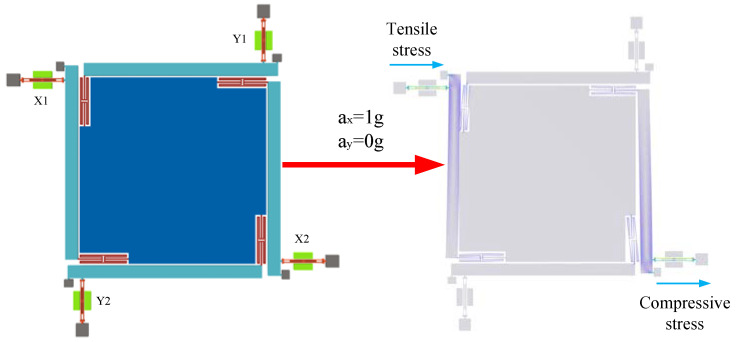
Biaxial resonant accelerometer working principle.

When acceleration is input along the *X*-axis, the proof block is oriented along the *X*-axis under the action of inertial forces. Since the decoupled structure has a large stiffness in the *X*-axis direction and a small stiffness in the *Y*-axis direction, the inertia force can be effectively transferred to the single-stage microlever mechanism. The inertial force is amplified by a single-stage microlever mechanism, and it is finally transferred to the resonator. The two resonators placed along the *X*-axis at both ends of the SRA are subjected to tensile and compressive stresses, respectively, and the resonance frequencies increase and decrease, respectively, as shown in Figure 2. The final acceleration is obtained by differential calculation.

### 2.1. Design of DETF Resonator with Coupled Structure

**Figure 3:**
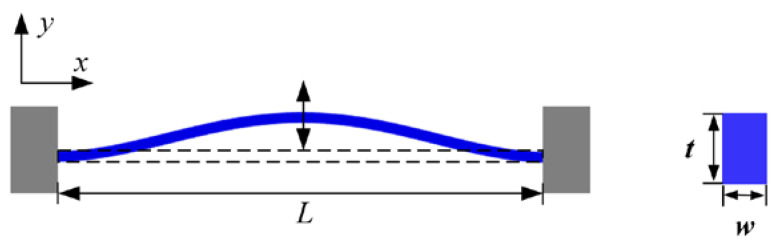
First-order mode shapes for bending vibration of double-ended solidly supported beams.

The conventional DETF resonator consists of two identical double-ended solidly supported beams, so the analysis of the DETF can be simplified to the model shown in Figure 3. The symmetric and antisymmetric modes of the DETF resonator correspond to the first-order bending modes of vibration of a double-ended solidly supported beam, and the dynamic equations for the undamped free vibration of a double-ended solidly supported beam under the action of an axial force are as follows:

(1) $$\mathrm{EI}\frac{\partial^{4}y}{\partial x^{4}}+F_{a}\frac{\partial^{2}y}{\partial x^{2}}+\rho S\frac{\partial^{2}y}{\partial t^{2}}=0$$

In Equation (1), y is the deflection of the beam, E is Young’s modulus, *ρ* is the density, I is the moment of inertia of the beam cross-section, S = tw is the area of the beam cross-section, t is the thickness of the beam, w is the width of the beam, and F_a_ is the axial force exerted on the beam.

The intrinsic frequency f_i_ of the i-th order mode of the resonator is the following:

(2) $$f_{i}=\frac{1}{2\pi }\sqrt{\frac{K_{i}}{M_{i}}}$$

In Equation (2), M_i_ is the equivalent mass of the resonator for the i-th order mode and K_i_ is the equivalent stiffness of the resonator for the i-th order mode.

When the DETF resonator is not subjected to any axial force, its first-order modal intrinsic frequency is the following:

(3) $$f_{i}=\frac{3.57}{L^{2}}\sqrt{\frac{EI}{\rho tw}}$$

When the DETF resonator is subjected to an axial force, it causes an increase or decrease in the resonant frequency with a frequency change Δf of the following [16]:

(4) $$\Delta f=f_{1}\left(\sqrt{1\pm \frac{0.3L^{2}}{Etw^{2}}F_{a}}-1\right)$$

In Equation (4), the ‘+’ and ‘−’ signs indicate that the resonance frequency increases and decreases when the resonator is subjected to tension and pressure, respectively.

**Figure 4:**
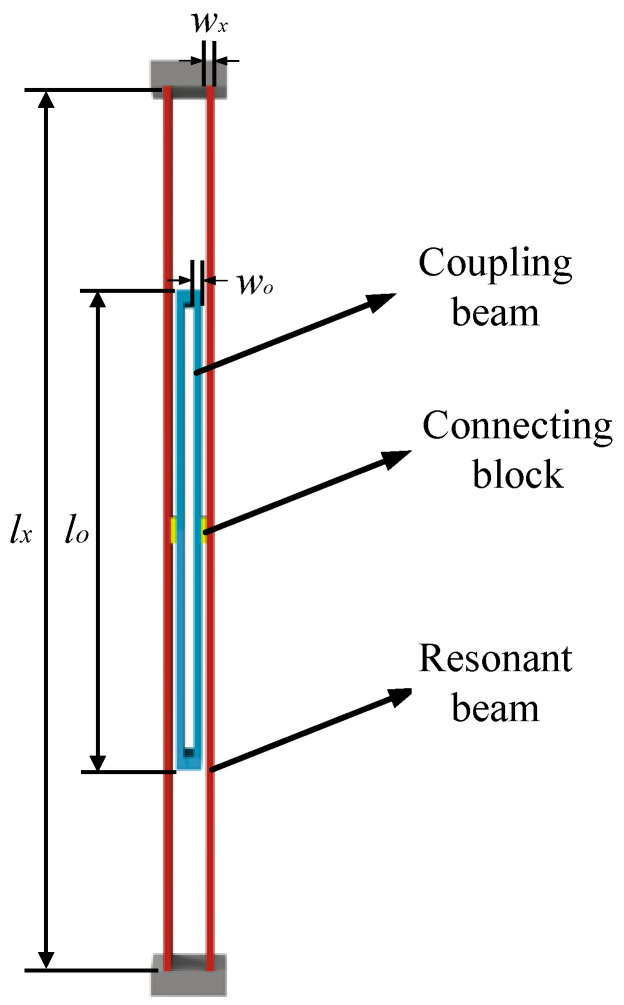
Schematic diagram of DETF resonator with coupling structure.

When a conventional DETF resonator operates in a symmetrical mode, the two resonant beams vibrate in opposite directions. The forces on the base cancel each other out, and the symmetric mode thus has a higher Q value than the antisymmetric mode. However, the intrinsic frequencies of the antisymmetric modes are lower than those of the symmetric modes, and the intrinsic frequencies of the two are similar. In practice, symmetric modes are easily converted to antisymmetric modes. To solve the above problems, in this paper, a coupling structure will be used in the DETF resonator, as shown in Figure 4.

When the DETF resonator with coupled structure operates in symmetric modes, since its modes are symmetric, the equivalent stiffness and mass are solved for only one of the sides. Since the dimensions of the connecting blocks are small relative to the resonator and coupling beams, they are neglected in the calculations. The equivalent mass of the resonator can be expressed by Equation (5) [13]:

(5) $$m=0.3714m_{x}+0.3714m_{o}$$

In Equation (5), m_x_ is the mass of a single resonant beam, and m_o_ is the mass of a single coupled beam.

In the second-order symmetric mode of the resonator, the stiffnesses of the resonant beam and the coupling beam are superposed, and the two satisfy the relation (6):

(6) $$k=k_{x}+k_{o}$$

In Equation (6), k_x_ is the equivalent stiffness of a single resonant beam, and k_o_ is the equivalent stiffness of a single coupled beam.

Bringing Equations (5) and (6) into Equation (2), the unilateral intrinsic frequency f_01_ of the resonator can be expressed by Equation (7):

(7) $$f_{01}=\frac{1}{2\pi }\sqrt{\frac{\frac{4Ew_{x}^{3}t}{l_{x}^{3}}+\frac{4Ew_{o}^{3}t}{l_{o}^{3}}}{0.3714m_{x}+0.3714m_{0}}}$$

In Equation (7), l_x_ is the length of the resonant beam, l_o_ is the length of the coupled beam, w_x_ is the width of the resonant beam, and w_o_ is the width of the coupled beam.

Conventional DETF resonators do not have a connection structure between the two sides of the resonator, but DETF resonators with a coupling structure have two sides of the resonator connected by a coupling structure. Thus, the two sides of the resonator form a whole through the coupling structure. The intrinsic frequency f_0_ of the DETF resonator with coupling structure can be expressed by Equation (8):

(8) $$f_{0}=\frac{1}{2\pi }\sqrt{\frac{\frac{16Ew_{x}^{3}t}{l_{x}^{3}}+\frac{16Ew_{o}^{3}t}{l_{o}^{3}}}{0.3714m_{x}+0.3714m_{0}}}$$

**Figure 5:**
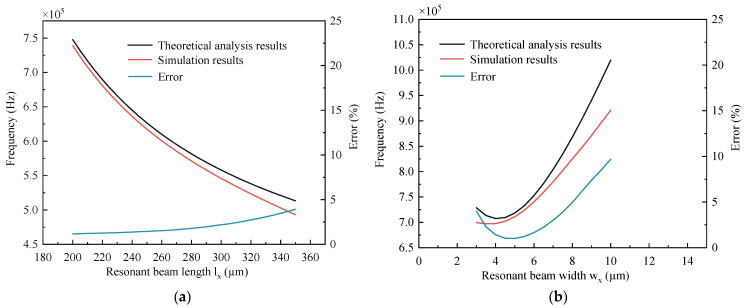

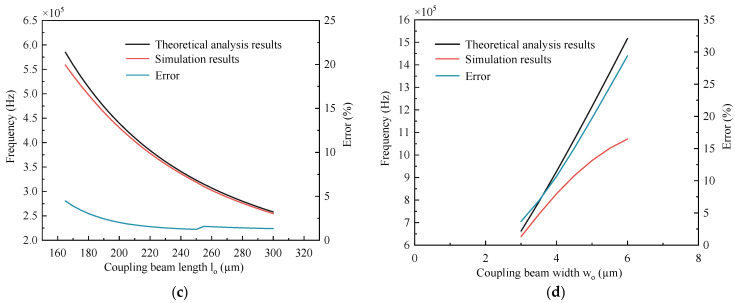
Comparison of theoretical and finite element analysis results: (**a**) Length of the resonant beam; (**b**) Width of the resonant beam; (**c**) Coupling beam length; (**d**) Coupling beam width.

The dimensions of the resonant and coupled beams are selected in a certain range, and the intrinsic frequency of the structure with coupling is calculated using the proposed mathematical model. The calculated data are compared with the data obtained from simulation using COMSOL 6.1 commercial software, and the results are shown in Figure 5.

From Figure 5a, it can be seen that when the length of the resonant beam increases, both the theoretical analysis results and the finite element analysis results show that the intrinsic frequency is decreasing. The error tends to increase with the length of the resonant beam, but the increase is small, and the error can be maintained in a small range. As can be seen from Figure 5b, when the width of the resonant beam increases, both the theoretical analysis results and the FEA results indicate that the intrinsic frequency is increasing. The error shows an increasing trend with the increase in the width of the resonant beam; the increase is large, and the error is minimal when the width of the resonant beam is 5 µm. From Figure 5c, it can be seen that when the length of the coupled beam increases, both the theoretical analysis results and the finite element analysis results indicate that the intrinsic frequency is decreasing. The error shows a decreasing trend as the length of the coupling beam increases, and the error can likewise be maintained in a small range. As can be seen from Figure 5d, when the width of the coupled beam increases, both the theoretical and FEA results indicate that the intrinsic frequency is increasing. The error shows an increasing trend with the increase in the width of the coupling beam; the increase is large, and the error is minimal when the width of the coupling beam is 3 µm.

The results In Figure 5 show that when the lengths of the resonant beam and the coupling beam vary within a certain range, the analysis can be carried out effectively using Equation (8). When the width of the resonant beam and the width of the coupling beam are too large, the error of the analysis using Equation (8) is larger. In the design of resonant accelerometers, the intrinsic frequency of the resonator cannot be too high; this ensures higher sensitivity and better detection. Therefore, the width of the resonator and coupling beam should not be too large. Equation (8) can provide an effective theoretical analysis for resonators within a certain size range.

**Figure 6:**
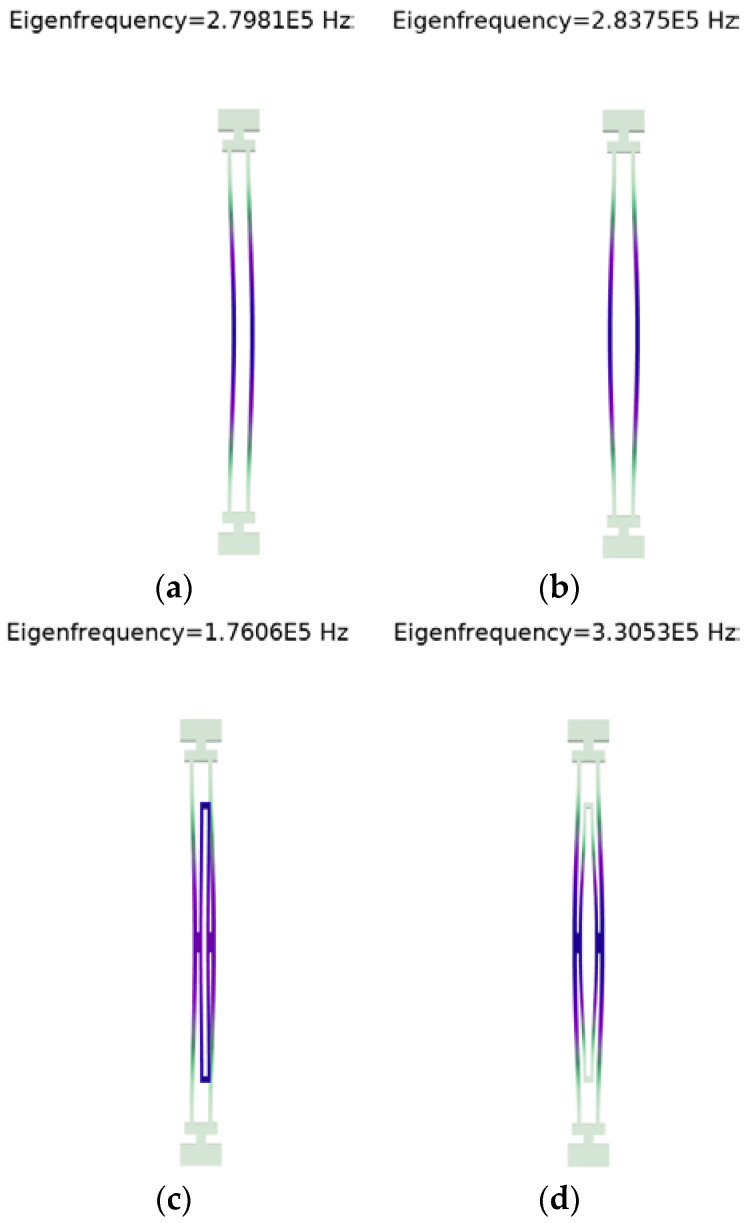
Comparison of resonance frequency between conventional DETF resonator and DETF resonator with coupling structure: (**a**) Conventional DETF resonator antisymmetric mode intrinsic frequency 279.810 kHz; (**b**) Conventional DETF resonator symmetrical mode intrinsic frequency 283.750 kHz; (**c**) DETF resonator with coupled structure antisymmetric mode intrinsic frequency 176.060 kHz; (**d**) Symmetric modal intrinsic frequency of DETF resonator with coupled structure 330.530 kHz.

The coupling structure can separate the resonant frequencies between the symmetric and antisymmetric modes, effectively avoiding the interference between the resonator itself and the external environment, reducing the base loss, and reducing the energy loss. The intrinsic frequencies of the conventional DETF resonator and the DETF resonator with coupling structure are shown in Figure 6.

As can be seen in Figure 6, the frequency difference between the symmetric and antisymmetric modes is changed from 3940 Hz to 154,470 Hz after adopting the coupling structure, which indicates that the coupling structure can effectively isolate the symmetric and antisymmetric modes, thus avoiding the modal interference of the resonator itself.

**Figure 7:**
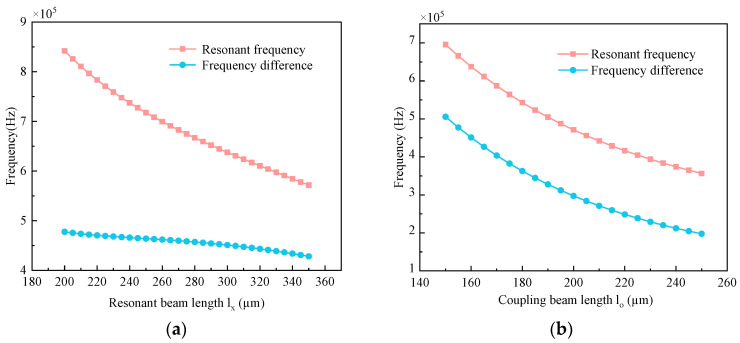
Effect of resonant beam length l_x_ and coupled beam length l_o_ on resonant frequency and frequency difference Δf: (**a**) Length of resonant beam l_x_; (**b**) Coupling beam length l_o_.

The coupling structure in a resonator consists of a coupling beam and a connecting block. Changing the length of the coupling beam and the resonant beam will change the resonant frequency of the symmetric modes, and the frequency difference between the symmetric and antisymmetric modes will also be changed. By varying the length l_x_ of the resonant beam and the length l_o_ of the coupling beam, the resonant frequency change of the resonator and the frequency difference change Δf are shown in Figure 7.

As shown in Figure 7a, the resonant frequency of the resonator decreases with the increase in the length l_x_ of the resonant beam, and the frequency difference Δf also decreases, but the trend is slower. As shown in Figure 7b, the resonant frequency of the resonator decreases with the increase in the coupling beam length l_o_, and the frequency difference Δf also decreases. Therefore, it is possible to change the resonant frequency of the resonator by changing the length of the coupling beam l_o_ without changing the length of the resonant beam l_x_, thus avoiding the influence of other interfering modes in the accelerometer.

### 2.2. Design of Decoupled Structures

**Figure 8:**
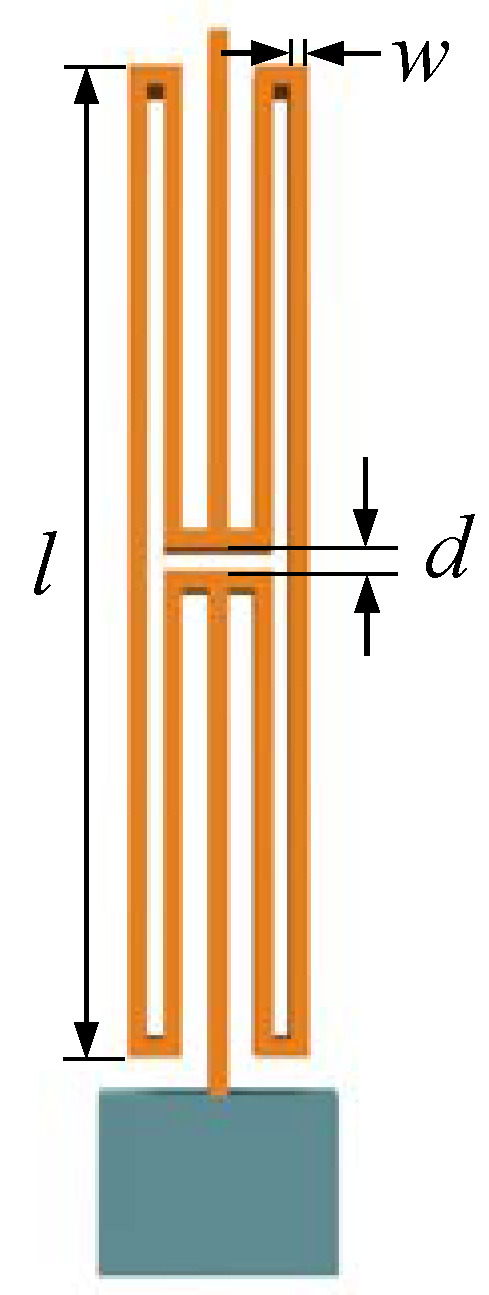
Schematic diagram of decoupling structure.

In the design of the MEMS resonant accelerometer, an H-beam is used as the decoupling structure, as shown in Figure 8. The decoupled beam is connected to the mass block at one end and to a single-stage microlever mechanism at the other end. The decoupling effect is achieved by the great difference between its axial stiffness k_y_ and transverse stiffness k_x_.

**Figure 9:**
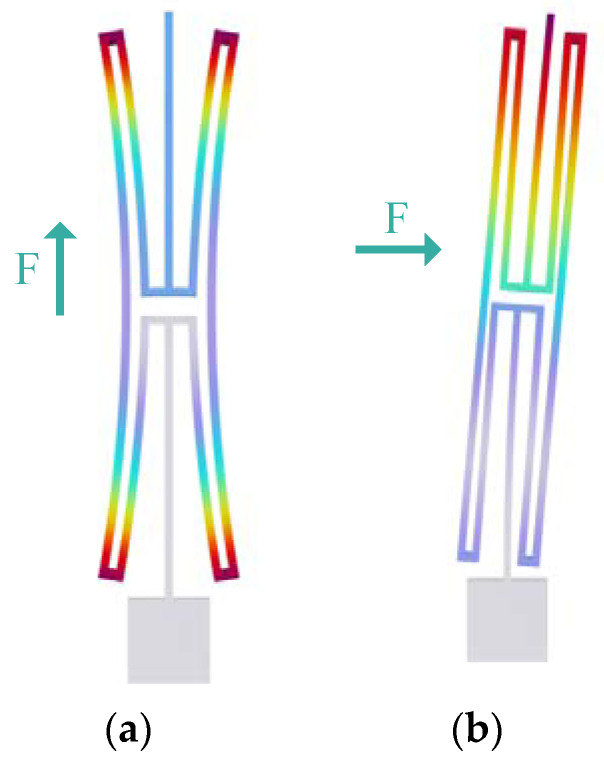
Finite element analysis results of decoupling structure axial stiffness and lateral stiffness: (**a**) Axial stiffness k_y_ = 5642.54 N/m; (**b**) Lateral stiffness k_x_ = 7.04 N/m.

To calculate the axial stiffness k_y_ and transverse stiffness k_x_ of the decoupled structure, finite element mechanical analysis based on the commercial software COMSOL is used. The deformation of the decoupled structure in the direction of the force is obtained by applying axial and transverse forces to the decoupled structure, respectively, and then the values of axial and transverse stiffness are obtained [20], as shown in Figure 9.

As shown in Figure 9, the axial stiffness k_y_ of the decoupled structure is 5642.54 N/m, and the lateral stiffness k_x_ is 7.04 N/m. The difference between the two is very large, which can effectively isolate the crosstalk between the sensitive and non-sensitive directions.

**Figure 10:**
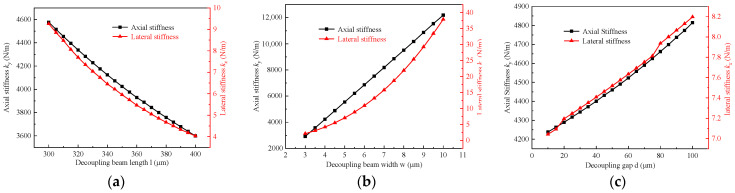
Influence of dimensional parameters of decoupled structures on axial and lateral stiffness: (**a**) Decoupling beam length; (**b**) Decoupling beam width; (**c**) Decoupling gap.

In order to analyze the influence of the dimensional parameters of the decoupling structure on the decoupling effect, three-dimensional parameters of the decoupling structure are extracted. They are decoupling beam length *l*, decoupling beam width *w*, and decoupling gap *d*. The effects of the above three-dimensional parameters on the axial stiffness k_y_ and transverse stiffness k_x_ are obtained by using COMSOL finite element analysis, as shown in Figure 10.

As shown in Figure 10a, the axial stiffness and lateral stiffness of the decoupled structure decrease with the increase in decoupled beam length. When the length of the decoupled beam changes from 300 µm to 400 µm, the axial stiffness decreases from 4576.66 N/m to 3598.41 N/m, and the transverse stiffness decreases from 9.26 N/m to 4.03 N/m. This shows that the length of the decoupled beam has a significant effect on the stiffness. As shown in Figure 10b, the axial and lateral stiffness of the decoupled structure increases with the increase in the width of the decoupled beam. When the width of the decoupled beam is varied from 3 µm to 11 µm, the axial stiffness is increased from 2913.75 N/m to 12,189.18 N/m, and the lateral stiffness is increased from 2.13 N/m to 37.87 N/m. This indicates that the width of the decoupled beam also has a significant effect on the stiffness. As shown in Figure 10c, the axial stiffness and lateral stiffness of the decoupled structure increase slightly with the increase in the decoupling gap. When the decoupling gap is varied from 10 µm to 100 µm, the axial stiffness increases from 4238.54 N/m to 4814.64 N/m, and the lateral stiffness increases from 7.04 N/m to 8.20 N/m. This indicates that the effect of the decoupling gap on stiffness is relatively small.

### 2.3. Design of Single-Stage Microlever Mechanism

**Figure 11:**
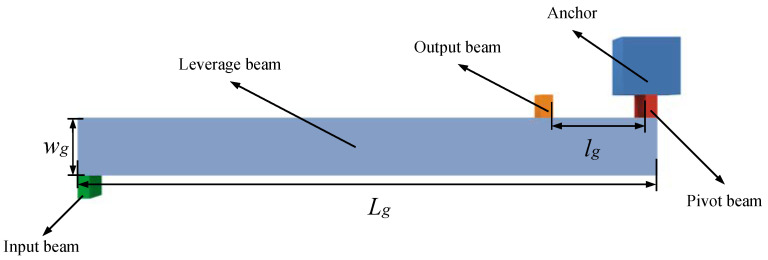
Schematic diagram of single-stage micro leverage mechanism.

The single-stage micro-lever mechanism used in this paper consists of an input beam, a lever beam, a pivot beam, and an output beam [21], as shown in Figure 11. Unlike conventional mechanical levers, the single-stage microlever mechanism for SRAs is a flexible lever in which the input beam is replaced by a decoupled structure and connected to a mass block. The pivot beam is connected to an anchor point, and the output beam is connected to one end of the resonator. The output beam is located between the pivot beam and the input beam, and the power arm is much larger than the resistance arm; this microlever mechanism has force amplification characteristics.

When an inertial force is applied to the input beam, the lever beam is displaced in the direction of the force by a certain amount x and undergoes a certain angular deflection *θ*. According to the principles of force equilibrium and moment equilibrium,

(9) $$F_{i}=F_{o}+F_{p}$$

(10) $$F_{i}L=F_{o}l+M_{p}+M_{r}+M_{c}$$

where F_i_, F_p_, and F_o_ are the axial forces in the input, pivot, and output beams, respectively. M_r_, M_p_, and M_c_ are the bending moments in the input, pivot, and output beams, respectively.

By analyzing the relationship between the bending moments of the input, output, and fulcrum beams and the corresponding axial and rotational stiffnesses, it can be deduced that the amplification of the single-stage microlever system is as follows [16]:

(11) $$A=\frac{\frac{1}{k_{vp}}\left(k_{\theta p}+k_{\theta i}+k_{\theta o}\right)+L_{g}\;l_{g}}{\left(\frac{1}{k_{vo}}+\frac{1}{k_{vp}}\right)\left(k_{\theta p}+k_{\theta i}+k_{\theta o}\right)+l_{g}^{2}}$$

In Equation (11), k_θp_, k_θi_, and k_θo_ are the rotational stiffnesses of the pivot beam, the input beam, and the output beam, respectively. K_vp_, k_vi_, and k_vo_ are the axial stiffnesses of the pivot beam, the input beam, and the output beam, respectively. L_g_ is the length of the power arm, and l_g_ is the length of the resistance arm.

In the ideal case, the magnification A can be expressed as follows:

(12) $$A=\frac{L_{g}}{l_{g}}$$

**Figure 12:**
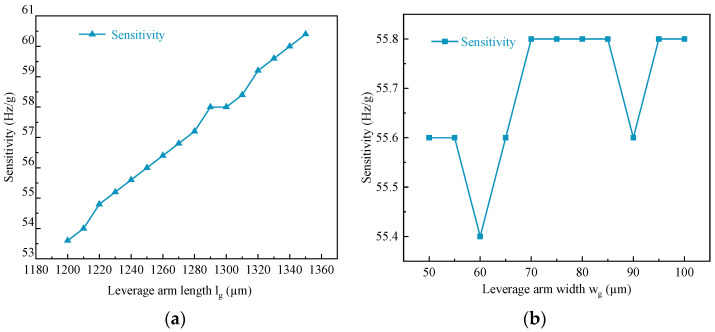
Effect of dimensional parameters of lever beam on sensitivity: (**a**) Lever beam length; (**b**) Lever beam width.

When the inertial force generated by the mass block is amplified by the single-stage micro-lever mechanism, it is applied to the resonator, which in turn changes the resonant frequency. The dimensional parameters of the single-stage micro-lever mechanism affect its amplification and, hence, the sensitivity of the accelerometer. By analyzing the influence of the dimensional parameters of the single-stage micro-lever mechanism on the sensitivity, the appropriate dimensional parameters can be effectively selected for structural design. The effect of the dimensional parameters of the lever beam on the sensitivity is shown in Figure 12.

The effect of the length l_g_ of the lever beam on the magnification A is shown in Figure 12a. It can be seen from the figure that as the length of the lever beam increases, the sensitivity gradually increases, and the relationship between the two is positively correlated. The effect of the width w_g_ of the lever beam on the magnification A is shown in Figure 12b. It can be seen from the figure that the sensitivity is constant as the width of the lever beam increases. This agrees with the findings of Ding et al. [21].

## 3. BP and NSGA-II Algorithm to Optimize Decoupled Structures

To ensure the performance of accelerometers with greater sensitivity and less cross-axis sensitivity, it is necessary to design the structure with greater axial stiffness and less lateral stiffness. However, the axial and lateral stiffnesses show the same trend as the decoupled beam parameters. When the parameters are chosen to satisfy high axial stiffness, the lateral stiffness also tends to be higher. The axial stiffness also tends to be lower when parameters satisfying low lateral stiffness are selected. This contradictory relationship implies that the single-objective optimization method cannot satisfy the need for designing an optimal structure. In such a case, the exhaustive method is usually used to find the optimal solution within a certain parameter range, but the exhaustive method is inefficient, the solution is difficult to obtain in a reasonable amount of time, and the method is not adapted to the dynamically changing problem environment. Therefore, it becomes challenging to obtain the optimal parameters of the decoupled structure. To solve the above problems, this paper proposes a multi-objective optimization scheme for the decoupled structure based on the BP algorithm and the NSGA-II algorithm to achieve the simultaneous optimization of the axial and lateral stiffnesses.

### 3.1. Optimization Principles and Processes

BP neural network has powerful nonlinear mapping ability, adaptive learning ability, and strong generalization ability, which can fit complex functional relationships and effectively predict and classify unknown data. It is also fault-tolerant and can handle partially missing or noisy data [22,23]. In addition, the BP neural network structure allows parallel processing, which can improve computational efficiency and is widely used in several fields. Through the backpropagation algorithm, BP neural networks can gradually approximate the optimal solution, allowing them to perform well when dealing with complex and diverse data. Therefore, BP neural networks are particularly suitable for fitting the unknown relationship between the dimensional parameters and the stiffness of decoupled structures.

The NSGA-II algorithm is characterized by fast, non-dominated ordering, efficient congestion distance computation, and elite policies. It significantly improves the computational efficiency and diversity maintenance of the solution, ensuring the retention of good individuals and the convergence performance of the algorithm. It is adaptable, capable of handling various types of multi-objective optimization problems without complex parameter tuning, and easy to use and implement [24].

As can be seen from Figure 10, the three-dimensional parameters of decoupling beam length l, decoupling beam width w, and decoupling gap d affect both axial stiffness k_y_ and transverse stiffness k_x_. To extract the parameter mapping relationship of the decoupled structure, a high-precision BP neural network is constructed. Three hundred sets of size combinations with different parameters were randomly extracted. The corresponding axial stiffness k_y_ and transverse stiffness k_x_ of each parameter combination were calculated using the commercial software COMSOL. Among them, 240 sets of data were selected as training sets and 60 sets of data as validation sets for training the BP neural network. Some of the data are shown in Table 1.

**Figure 13:**
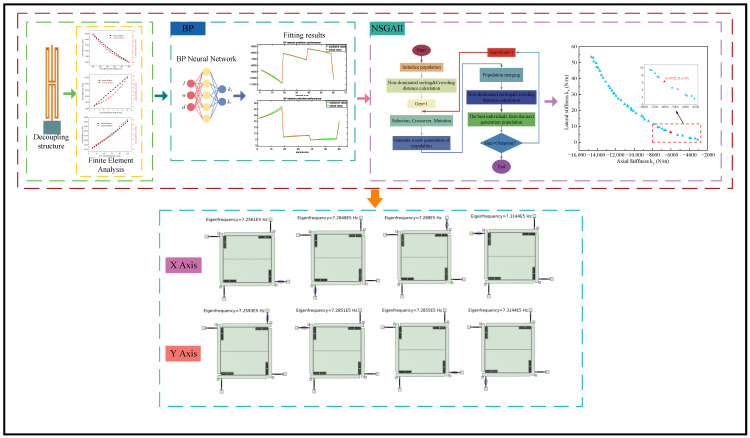
Decoupled structural optimization frameworks.

Based on the dataset generated by the FEA method, it is introduced into the BP training network as a training and testing set. A BP neural network with three inputs and two outputs is constructed using decoupled beam length l, decoupled beam width w, and decoupling gap d as input independent variables and axial stiffness k_y_ and transverse stiffness k_x_ as output dependent variables. The functional relationship fitted by the BP neural network was used as the objective function for the iterative optimization of the NSGA II algorithm. The Pareto solution set with two outputs after 500 iterations of the population is taken as the optimal solution set. The most compliant solution in the optimal solution set is selected as the optimal structure parameter. The optimization framework of the decoupled structure is shown in Figure 13.

BP neural network contains input, hidden, and output layers. Each layer has corresponding weights and thresholds. The BP neural network structure used in this paper consists of three input parameters and two output parameters. The hidden layer has seven nodes. The input and output matrices are shown in Equations (13) and (14).

(13) $$X=\left[\begin{array}{ccc} l & w & d \end{array}\right]$$

(14) $$Y=\left[\begin{array}{cc} k_{y} & k_{x} \end{array}\right]$$

To increase the training speed in BP neural networks, stabilize the training process, prevent numerical overflow, improve model performance, and simplify weight initialization. Therefore, all data are normalized. Normalization can scale the data to a smaller range, reducing the problems of gradient explosion and gradient vanishing. And it makes it easier for the model to find the optimal solution and enhances the generalization ability, which leads to better performance on the test set. The normalization process is shown in Equations (15) and (16) [18].

(15) $$X_{i}=2\times \frac{x_{i}-x_{min}}{x_{max}-x_{min}}-1\left(i=1,2,\cdots ,n\right)$$

(16) $$Y_{i}=\frac{\left(y_{i}+1\right)\times \left(y_{max}-y_{\min}\right)}{2}+y_{\min}\left(i=1,2,\cdots ,n\right)$$

In the BP network model, the iterative adjustment of the weights is carried out through the error backpropagation algorithm. When the root mean square error between the fitted output data of the model and the actual value exceeds the expected value of the current iteration, the error backpropagation algorithm is used for adjustment. The error signal is propagated backward layer by layer, starting from the output layer. The weights and thresholds of each layer are gradually adjusted to reduce the output error by calculating the error contribution of neurons in each layer. This adjustment process continues until the rms error is gradually reduced to within the desired accuracy. The root mean square error is calculated as shown in Equation (17) [18].

(17) $$E=\frac{1}{n}\sum_{i=1}^{n}{\left(g_{i}-{\hat{g}}_{i}\right)}^{2}$$

In Equation (17), n is the number of samples, $g_{i}$ is the actual value, and ${\hat{g}}_{i}$ is the predicted value of the model. By continuously iterating and adjusting the weights and thresholds, the BP network model can optimize the performance and make the prediction results more accurate.

**Figure 14:**
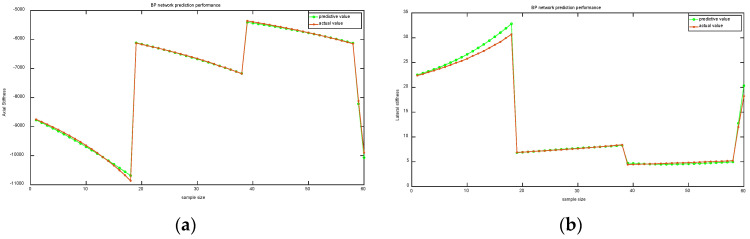
BPNN fitting results: (**a**) Axial stiffness; (**b**) Lateral stiffness.

A BP neural network was used to fit the data, and the running time of the program was 0.43463 s. The results of the BP neural network fitting are shown in Figure 14.

As can be seen from Figure 14, after the BPNN fitting, the fitted curves of axial stiffness and transverse stiffness have a high degree of overlap with the actual curves, which indicates that the BPNN fitting results have a small error and the BPNN fitting results have a high degree of validity.

The NSGA-II algorithm can achieve effective solutions to multi-objective optimization problems by combining non-dominated sorting and elite strategies. Non-dominated sorting divides the individuals in the population into multiple frontier levels by non-dominated sorting according to their objective function values. The first frontier level contains all non-dominated individuals, the second frontier level contains individuals dominated only by individuals in the first frontier level, and so on.

For individuals i and j, if i is dominated by j, it is satisfied:

(18) $$\forall k\in \left\{1,2,\cdots ,M\right\},f_{k}\left(i\right)\geq f_{k}\left(j\right)$$

(19) $$\exists k\in \left\{1,2,\cdots ,M\right\},f_{k}\left(i\right)>f_{k}\left(j\right)$$

where M is the number of objective functions and f_k_ denotes the kth objective function.

For each objective function, the crowding distance z_i_ for individual i is calculated as shown in Equation (20):

(20) $$z_{i}=\sum_{k=1}^{M}\frac{f_{i+1}^{k}-f_{i-1}^{k}}{f_{\max}^{k}-f_{\min}^{k}}$$

In Equation (20), $f_{i+1}^{k}$ and $f_{i-1}^{k}$ are the target values of the neighboring individuals of individual i on target f_k_, and $f_{\max}^{k}$ and $f_{\min}^{k}$ are the maximum and minimum values of f_k_, respectively.

The elite selection strategy of NSGA-II ensures that the optimal individuals in each generation are not lost by combining the parent and offspring populations for non-dominated sorting and crowding distance calculation. This strategy preserves the optimal individuals in each generation and prevents excellent solutions from being lost during the evolutionary process. This strategy accelerates the convergence rate and improves the quality of the solutions while maintaining the diversity of the population. The non-dominated ordering and congestion distance computation play an important role in maintaining diversity by preventing the algorithm from prematurely converging to a local optimum solution, which makes NSGA-II perform well in dealing with complex multi-objective optimization problems.

**Figure 15:**
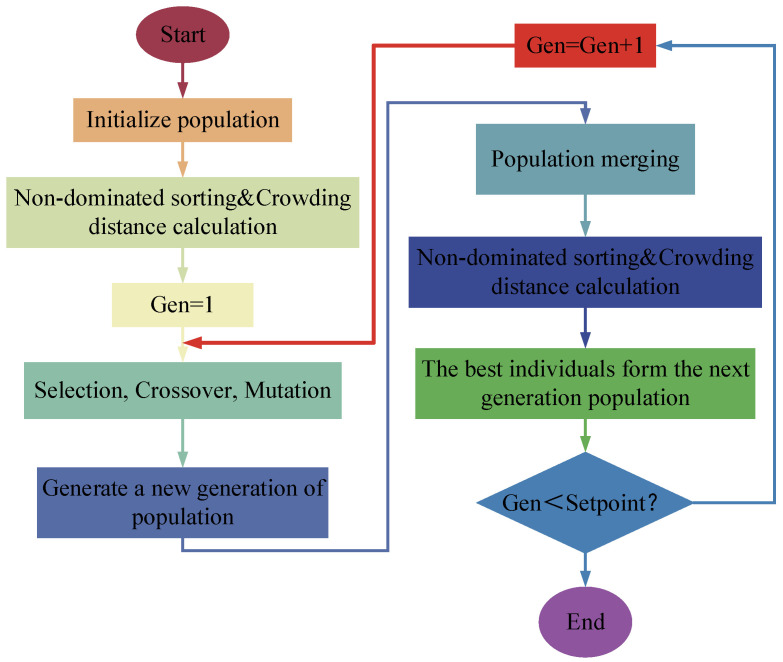
Optimization flowchart for NSGA-II algorithm.

The optimization flow of the NSGA-II algorithm is shown in Figure 15. First, the initial population P is generated; then, the population is non-dominatedly sorted, and the crowding distance z_i_ of each individual is calculated. Next, the individuals are selected into the selection pool based on the non-dominated sorting and the crowding distance z_i_; the crossover and mutation operations are performed on the individuals in the selection pool to generate the new generation of the population Q_t_; the parent generation of the population P_t_ and the offspring of the generation Q_t_ are merged to form the joint population R_t_, and the merged population R_t_ is selected by non-dominated sorting and crowding distance calculation. For non-dominated sorting and congestion distance calculation, select the optimal individual to form the next generation population P_t+1_; repeat the above steps until the termination condition is satisfied. Through this process, NSGA-II effectively maintains the diversity and optimization direction of the populations.

In order to prevent the results from not converging during the optimization process due to some parameters being large and to avoid the structure being unworkable or difficult to machine due to some parameters being too small, the three objective parameter boundaries of the optimization are constrained. The constraints are shown in Equation (21).

(21) $$\left\{\begin{array}{c} 200\;µm\leq l\leq 350\;\mu m\\ 3\;µm\leq w\leq 10\;µm\\ 5\;µm\leq d\leq 50\;µm \end{array}\right.$$

### 3.2. Optimization Results

The number of iterations is often used as one of the termination conditions when optimizing with the NSGA-II algorithm. By setting the number of iterations, the computational time and resource consumption of the algorithm can be controlled while ensuring the quality and diversity of the solutions. NSGA-II will gradually evolve closer to the Pareto frontier during the iterative process. As the number of iterations increases, the solutions in the population will gradually approach the global optimal solution set, and the quality of the solutions will gradually improve. However, the improvement effect of an algorithm tends to diminish as the number of iterations increases. By setting the number of iterations, the algorithm can be terminated at a reasonable level of convergence, thus avoiding unnecessary computational consumption. In some cases, several iterations that are too long may cause the algorithm to overfit some specific objective function features, leading to a decrease in the diversity of solutions. Setting a suitable number of iterations can avoid this and maintain the diversity of the solution set. By taking the optimization process of Lv et al. [18] and Zhang et al. [19] as a reference and improving the optimization efficiency based on it, the determined number of iterations is 500.

**Figure 16:**
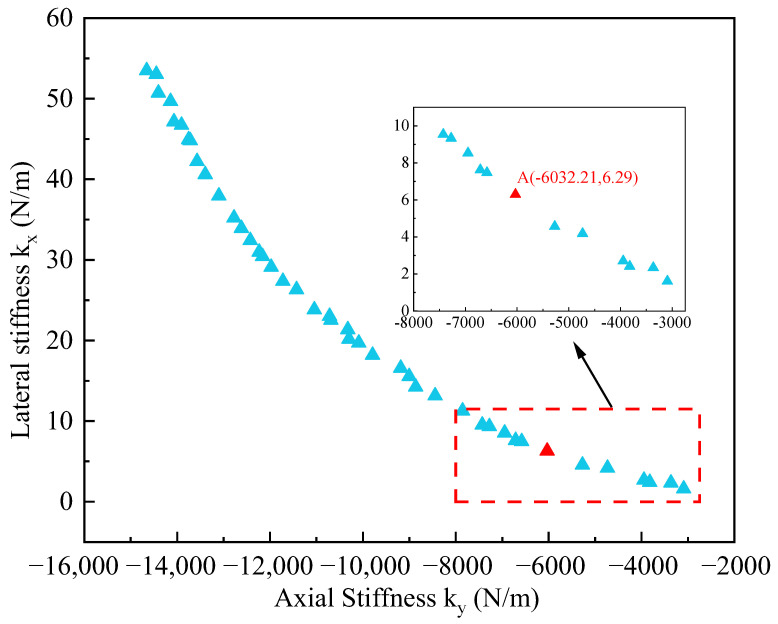
Pareto optimal solution set.

After 500 iterations using the NSGAII algorithm, the program runs in 146.2339 s, and the set of Pareto optimal solutions obtained is shown in Figure 16. The Pareto optimal solution set obtained after optimization by the BP-NSGAII algorithm is shown in Figure 15. The horizontal axis represents the axial stiffness k_y_ of the decoupled structure, and the vertical axis represents the lateral stiffness k_x_ of the decoupled structure. To improve the performance of the biaxial resonant accelerometers and to better select the appropriate dimensional parameters, the axial stiffness and lateral stiffness of the decoupled structure designed by Ding et al. are used as references. The decoupled structure with axial stiffness k_y_ greater than 2619.4 N/m and lateral stiffness k_x_ less than 11.4 N/m is selected [20]. The red-boxed part in Figure 16 shows the selection results.

For the overall structure of a resonant accelerometer, the operating frequency should be separated from the interference frequency. The dimensional parameters of the decoupled structure are likewise a key factor influencing the interference frequency. The frequency difference between the operating frequency and the interference frequency in the resonant accelerometer designed by Niu et al. is 7135.9 Hz [25]. In summary, in order to avoid the interference frequency close to the operating frequency resulting in a decrease in the measurement accuracy of the accelerometer, point A is selected as the optimal structural parameter. At this point, the length l of the decoupled beam is 313 µm, the width w is 5 µm, and d is 20 µm. The corresponding axial stiffness k_y_ of the decoupled structure is 6032.21 N/m, and the transverse stiffness k_x_ is 6.29 N/m. To ensure that the optimization results are accurate after 500 iterations, after obtaining the structural dimensional parameters given by the optimization results, the accuracy of the optimization results is verified by 3D modeling and finite element analysis using the commercial software COMSOL. The axial stiffness k_y_ obtained from the finite element analysis is 6029.54 N/m, and the transverse stiffness k_x_ is 6.25 N/m. Comparing the commercial software COMSOL finite element analysis results with the optimization results of the BP-NSGAII algorithm, the errors of the axial stiffness and transverse stiffness results are 0.0443% and 0.6359%, respectively. The finite element analysis results are in good agreement with the NSGA-II multi-objective optimization results, indicating the accuracy of the optimization results.

## 4. Overall Performance Analysis of Accelerometer

Based on the theoretical analysis of DETF resonators with coupled structures and single-stage micro lever mechanisms in Section 2, as well as the optimization results of the BP-NSGAII algorithm in Section 3, the specific dimensional parameters of the dual axis resonant MEMS accelerometer can be obtained, as shown in Table 2. The material used for the accelerometer is monocrystalline silicon, and the material’s properties are shown in Table 3.

### 4.1. Modal Analysis

**Figure 17:**
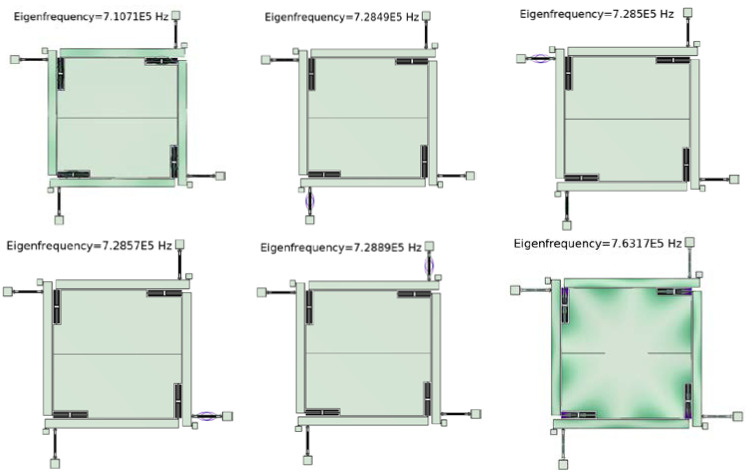
Operating modes and interference modes of SRAs.

After constructing a three-dimensional model of the accelerometer based on the size parameters in Table 2, modal analysis was performed using the commercial software COMSOL6.1. The working modes and interference modes of SRAs are shown in Figure 17. From the figure, it can be seen that the frequency difference between the working mode and the interference mode is 17,780 Hz and 34,280 Hz, and the interference mode is mainly the vibration mode of the decoupling structure. This indicates that the decoupling structure parameters must be selected so that the interference modes are far away from the working modes, which is consistent with the above point. From Figure 17, it can be seen that the choice of decoupling structure parameters is reasonable, and the accelerometer can effectively prevent the interference of other modes in the working process.

### 4.2. Impact Resistance Analysis

**Figure 18:**
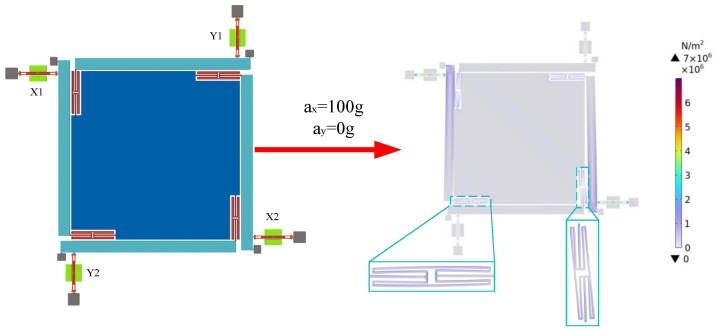
Accelerometer stress distribution after applying 100 g acceleration.

After applying a 100 g acceleration in the sensitive direction (*X*-axis), the displacement change of the accelerometer is shown in Figure 18. As shown in the figure, the stress distribution of the resonator in the *Y*-axis direction is much lower than that in the *X*-axis direction, indicating that the optimized decoupling structure effectively suppresses the transfer of inertial force in the non-sensitive axis direction. After applying an acceleration of 100 g, the maximum stress of the accelerometer is 7 × 10^6^ N/m^2^, which is much lower than the yield stress of silicon, 7 GPa, indicating that the designed accelerometer has a strong shock resistance.

### 4.3. The Impact of Environmental Factors

Environmental factors such as humidity, electromagnetic interference, and temperature can affect the performance of MEMS resonant accelerometers. A study by Huang et al. noted that common temperature and humidity variations result in a bandwidth decrease of about 25%, with negligible effects on Young’s modulus and coefficient of thermal expansion of conventional materials [26]. In their article, Tang et al. pointed out that electromagnetic interference can lead to device failures and safety hazards [27]. A study by Yin et al. pointed out that thermal stresses resulting from changes in external temperature can lead to frequency shifts and affect the performance of accelerometers [28].

To avoid the effect of temperature on the performance of the accelerometer, the accelerometer is designed to counteract the effect of temperature by using the measure of differential placement of the resonator. Taking the resonators X1 and X2 in Figure 2 as an example, when the accelerometer senses the external acceleration, the inertia force will be transferred to the resonators X1 and X2 through the decoupling structure and the lever structure. The resonant frequency f_X1_ of the resonator X1 under tensile stress varies, as shown in Equation (22). The resonant frequency f_X2_ of the resonator X2 under compressive stress varies, as shown in Equation (23).

(22) $$f_{X1}=f_{0}+\Delta f_{F}$$

(23) $$f_{X2}=f_{0}-\Delta f_{F}$$

where f_0_ is the intrinsic frequency of the resonator and Δf_F_ is the frequency change due to external acceleration.

When the resonator is affected by the external temperature, the resonant frequencies of resonators X1 and X2 will increase simultaneously. When the resonator is affected by the external acceleration and temperature at the same time, the resonant frequency f_1_ of resonator X1 and the resonant frequency f_2_ of resonator X2 are shown in Equations (24) and (25).

(24) $$f_{1}=f_{X1}+\Delta f_{T}$$

(25) $$f_{2}=f_{X2}+\Delta f_{T}$$

where Δf_T_ is the frequency change due to the external temperature.

To avoid the effect of temperature on the sensor, the resonance frequencies of resonators X1 and X2 can be subtracted from each other when calculating the sensitivity of the accelerometer. The sensitivity S of the accelerometer is shown in Equation (26), from which it can be seen that with the resonator set up differentially, the sensitivity is only related to the external inertial force.

(26) $$S=2\times \Delta f_{F}$$

To avoid the effects of humidity on the accelerometer, vacuum encapsulation, and waterproof coating are used when subsequently encapsulating the accelerometer. To avoid the influence of electromagnetic interference on the accelerometer, it is encapsulated in a metal tube housing. A low-pass filter and a high-pass filter are used during testing to filter out high-frequency electromagnetic interference and low-frequency noise signals to ensure the accuracy of the accelerometer’s output signal.

### 4.4. Sensitivity and Cross-Axis Sensitivity Analysis

**Figure 19:**
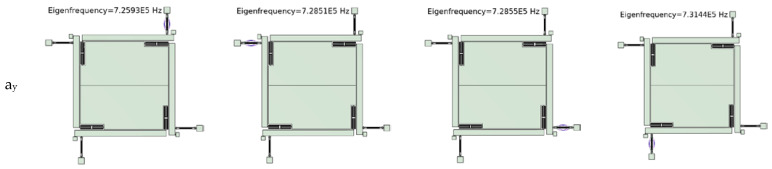

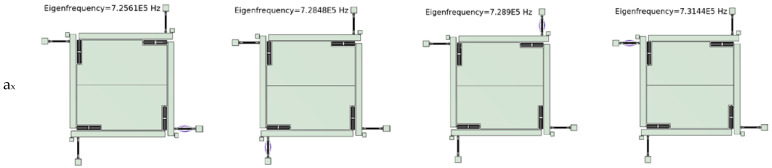
The resonance frequency of the accelerometer after applying 100 g acceleration to the *x*-axis and *y*-axis, respectively.

After applying 100 g of acceleration in the *Y*-axis direction, the resonance frequency of the Y1 resonator changes to 2960 Hz, the frequency of the Y2 resonator changes to 2950 Hz, the resonance frequency of the X1 resonator changes to 10 Hz, and the frequency of the X2 resonator changes to 20 Hz. After applying 100 g of acceleration in the *X*-axis direction, the resonance frequency of the Y1 resonator changes to 10 Hz, the resonance frequency of the Y2 resonator changes to 2960 Hz, the frequency of the X1 resonator changes to 10 Hz, and the frequency of the X2 resonator changes to 20 Hz. The frequency changes to 10 Hz, the resonance frequency of the X1 resonator changes to 2940 Hz, and the frequency of the X2 resonator changes to 2960 Hz, as shown in Figure 19. The sensitivity of the accelerometer is 59.1 Hz/g (*Y*-axis) and 59 Hz/g (*X*-axis); the cross-axis sensitivity is 0.508% (*Y*-axis) and 0.339% (*X*-axis). Compared with most current resonant accelerometers [15,16,25,29], the sensitivity of the accelerometer designed in this paper is slightly lower, but the parameter of cross-axis sensitivity is significantly better than other resonant accelerometers. Compared with Ding et al., who adopted the same decoupling structure, the cross-axis sensitivity of the *Y*-axis decreased by 85.1%, and the cross-axis sensitivity of the *X*-axis decreased by 90.03% [16]. This further demonstrates the effectiveness of the BP-NSGAII algorithm proposed in this article.

The designed accelerometer cross-axis sensitivity in this article was obtained by finite element analysis, and some factors (processing conditions, experimental environment, etc.) were not taken into account compared to the experimental results. This situation may lead to some discrepancies between the results of the subsequent experiments and the results of the finite element analysis. However, the cross-axis sensitivity of the accelerometer in this article is 0.508% (*Y*-axis) and 0.339% (*X*-axis). The *Y*-axis cross-axis sensitivity is reduced by 85.1%, and the *X*-axis cross-axis sensitivity is reduced by 90.03% [16], which is an advantage compared to Ding et al., who used the same decoupling structure. Therefore, although the errors in the experimental and processing processes may lead to some differences between the experimental results and the FEA results, such differences have less impact on the credibility of the FEA results. This article aims to show that the cross-axis sensitivity of accelerometers can indeed be effectively reduced after optimizing the decoupling structure using the BP-NSGAII algorithm. It can also be shown that optimizing the decoupling structure by the BP-NSGAII algorithm is an effective method in the design of resonant accelerometers. This approach provides a new way of thinking about solving design problems. To enhance the reliability and persuasiveness of the results in this article, at the current stage, the difference in data sources between the simulation results and the experimental results will be indicated in the article, as shown in Table 4.

## 5. Conclusions

To improve the measurement accuracy and stability of resonant accelerometers for automotive airbags, seismic monitoring, and navigation applications, the coupling structure is introduced based on the conventional DETF resonator, which effectively increases the frequency difference between symmetric and antisymmetric modes to avoid Q value loss. To reduce the cross-axis sensitivity more effectively while ensuring the measurement accuracy and reliability of the accelerometer, a multi-objective optimal design method for resonant accelerometers based on BP and NSGA-II algorithms is proposed. The solution optimizes the parameters of the decoupled structure in the accelerometer and effectively improves the difference between the axial and lateral stiffness of the decoupled structure. The optimization results were validated using the commercial software COMSOL, and the errors were 0.0443% and 0.6359%, respectively, indicating the high accuracy of the optimization results. The sensitivity of the designed biaxial SRA is 59.1 Hz/g (*Y*-axis) and 59 Hz/g (*X*-axis). The cross-axis sensitivity is 0.508% (*Y*-axis) and 0.339% (*X*-axis), which is significantly lower than other resonant accelerometers. The proposed multi-objective optimization algorithm for the decoupled structure of resonant accelerometers can also be used to optimize multiple parameters in the structure of other MEMS sensors to improve their structural performance.

## Figures and Tables

**Table 1:** BP Neural Network Partial Training Set.

*l*/µm	*w*/µm	*d*/µm	*k_y_*/N·m^−1^	*k_x_*/N·m^−1^
255	5	10	7090.187181	11.07419712
260	5	10	6973.500697	10.47120419
330	5	10	5681.172594	5.291005291
335	5	10	5604.75283	5.050505051
245	4	10	5543.237251	7.092198582
250	4	10	5452.562704	6.711409396
285	6	10	7977.026165	12.80409731
290	6	10	7857.92865	12.18026797
300	5	35	6397.952655	7.299270073
300	5	40	6447.453256	7.352941176
250	5	35	7515.971439	12.40694789
250	5	40	7584.951456	12.56281407
350	5	25	5495.109353	4.566210046
350	5	30	5533.115697	4.587155963
350	5	35	5568.548836	4.62962963
…	…	…	…	…

**Table 2:** Specific dimensional parameters of biaxial resonant MEMS accelerometers.

Parametric	Notation	Value
Proof of mass area	*S_proof_*	1.25 × 10^−6^ m^2^
Thicknesses	*t*	35 µm
Decoupling beam length	*l*	313 µm
Decoupling beam width	*w*	5 µm
Decoupling gap	*d*	20 µm
Lever beam length	*l_g_*	1250 µm
Lever beam width	*w_g_*	80 µm
Resonant beam length	*l_x_*	250 µm
Coupling beam length	*l_o_*	160 µm

**Table 3:** SRA material parameters.

Material	Young’s Modulus (Pa)	Poisson’s Ratio	Density (kg/m^3^)	CTE (1/K)	Thermal Conductivity (W/(m·K))
monocrystalline silicon	170 × 10^9^	0.28	2329	2.6 × 10^−6^	130

**Table 4:** Comparison between this work and others reported.

Reference	Weight of the Mass (kg)	Thickness (µm)	Resonant Frequency (kHz)	Sensitivity (Hz/g)	Cross-Axis Sensitivity	Source of Results
This work	1.019 × 10^−7^	35	744	59.1 Hz/g (*Y*-axis) 59 Hz/g (*X*-axis)	0.508% (*Y*-axis) 0.339% (*X*-axis)	Simulation
Yang et al. [15]	3.76 × 10^−8^	\	24 (*Y*-axis) 27 (*X*-axis)	51.64 Hz/g (*Y*-axis) 52.57 Hz/g (*X*-axis)	1.33% (*Y*-axis) 1.08% (*X*-axis)	Experiment
Ding et al. [16]	5.825 × 10^−8^	25	290	275 Hz/g	3.4%	Experiment
Niu et al. [25]	1.24 × 10^−6^	\	62	12.24 Hz/g (*Y*-axis) 21.30 Hz/g (*X*-axis)	1.55% (*Y*-axis) 1.92% (*X*-axis)	Experiment
Wang et al. [29]	1.84 × 10^−7^	\	\	1153.3 Hz/g	1.33%	Experiment

## Data Availability

The original contributions presented in the study are included in the article. Further inquiries can be directed to the corresponding author.
